# A neuro-cardiac self-regulation therapy to improve autonomic and neural function after SCI: a randomized controlled trial protocol

**DOI:** 10.1186/s12883-021-02355-w

**Published:** 2021-08-26

**Authors:** Ashley Craig, Ilaria Pozzato, Mohit Arora, James Middleton, Dianah Rodrigues, Candice McBain, Yvonne Tran, Glen M. Davis, Bamini Gopinath, Annette Kifley, Andrei Krassioukov, Jeffrey Braithwaite, Rebecca Mitchell, Sylvia M. Gustin, Jacob Schoffl, Ian D. Cameron

**Affiliations:** 1 John Walsh Centre Rehabilitation Research, Northern Sydney Local Health District, The Kolling Institute, Northern Clinical School, Faculty of Medicine and Health, The University of Sydney, St Leonards, NSW 2065 Australia; 2 Macquarie University Hearing (MU Hearing), Macquarie University, North Ryde, NSW 2113 Australia; 3 Exercise and Sports Sciences, Faculty of Medicine and Health, The University of Sydney, Sydney, NSW Australia; 4 ICORD, Faculty of Medicine, University of British Columbia, Vancouver, British Columbia V5Z 1M9 Canada; 5 Centre for Healthcare Resilience and Implementation Science, Australian Institute of Health Innovation, Health Systems Research, Macquarie University, North Ryde, NSW 2113 Australia; 6 School of Psychology, Faculty of Science, University of New South Wales, Kensington, NSW Australia

**Keywords:** Respiration, Electrocardiography, Electroencephalography, Near infra-red spectroscopy, heart rate variability feedback, cognitive impairment, mood disorder, autonomic nervous system, spinal cord injury.

## Abstract

**Background:**

Spinal cord injury (SCI) is associated with autonomic imbalance and significant secondary conditions, including cardiac and brain dysfunction that adversely impact health and wellbeing. This study will investigate the effectiveness (intention-to-treat) of a neuro-cardiac self-regulation therapy to improve autonomic and neural/brain activity in adults with SCI living in the community.

**Methods:**

A two-arm parallel, randomised controlled trial in which adults with SCI living in the community post-rehabilitation will be randomly assigned to a treatment or control group. The treatment group (*N* = 60) aged 18–70 years with a chronic traumatic or non-traumatic SCI, will receive intervention sessions once per week for 10 weeks, designed to regulate autonomic activity using computer-based feedback of heart rate variability and controlled breathing (called HRV-F). Comprehensive neurophysiological and psychological assessment will occur at baseline, immediate post-treatment, and 6 and 12-months post-treatment. Primary outcome measures include electrocardiography/heart rate variability (to assess autonomic nervous system function) and transcranial doppler sonography (to assess cerebral blood circulation in basal cerebral arteries). Secondary outcomes measures include continuous blood pressure, electroencephalography, functional near-infrared spectroscopy, respiration/breath rate, electrooculography, cognitive capacity, psychological status, pain, fatigue, sleep and quality of life. Controls (*N* = 60) will receive usual community care, reading material and a brief telephone call once per week for 10 weeks and be similarly assessed over the same time period as the HRV-F group. Linear mixed model analysis with repeated measures will determine effectiveness of HRV-F and latent class mixture modelling used to determine trajectories for primary and selected secondary outcomes of interest.

**Discussion:**

Treatments for improving autonomic function after SCI are limited. It is therefore important to establish whether a neuro-cardiac self-regulation therapy can result in improved autonomic functioning post-SCI, as well as whether HRV-F is associated with better outcomes for secondary conditions such as cardiovascular health, cognitive capacity and mental health.

**Trial registration:**

The study has been prospectively registered with the Australian and New Zealand Clinical Trial Registry (ACTRN12621000870853.aspx).

Date of Registration: 6th July 2021.

Trial Sponsor: The University of Sydney, NSW 2006.

Protocol version: 22/07/2021.

## Background

Spinal cord injury (SCI) is associated with damage to the nervous system and spinal cord as a result of bruising, compression or severance following a traumatic injury [1], or with deterioration of the spine, infection, vascular accident, or tumours in non-traumatic disease [2]. People with SCI also have higher risks of secondary health conditions, including sleep disorder [3, 4], psychological distress [5, 6], chronic pain [6, 7], elevated fatigue [8], cognitive impairment [9, 10] cardiovascular morbidity [1, 11] and autonomic nervous system dysfunction [1, 4, 11]. Dysfunctional autonomic activity after SCI impacts various bodily functions, including cardiac and brain function. However, treatment options and research targeting autonomic dysfunction after SCI are still limited.

Recently, there has been focus on restoring autonomic function in people with SCI using transcutaneous or surgically implanted epidural stimulation technology of the thoracolumbar cord [12, 13]. Preliminary studies have shown favourable results, such as temporary improvements in cardiovascular and motor function [12, 13]. However, there has been scarcely any research investigating benefits of non-invasive neuro-cardiac self-regulation interventions that could enhance neuroplasticity and improve autonomic/neural function following a SCI. Neuro-cardiac self-regulation (HRV-F) usually involves participants learning to regulate their autonomic neuro-cardiac activity via computer/mobile app-based feedback of heart rate variability (HRV) combined with instruction on slow/diaphragmatic breathing, with sessions delivered once per week for between 6 and 10 weeks [14]. Research has shown such neuro-cardiac self-regulation therapy to be a promising treatment for anxiety, depressive mood and trauma-related distress [15], asthma [16], pain [17], and heart disease [18].

The presumed mechanism for benefits associated with self-regulation of the autonomic nervous system involves a combination of physiological processes resulting in strengthening of baroreflex homeostasis, vagal afferent pathways, and phase relationships between blood pressure, heart-rate and brain connectivity [19–22]. Optimal autonomic regulation is associated with healthy functioning of the body’s major systems, particularly cardiac and brain function (e.g., cognitive and psychological aspects) [11, 19–22]. Following a SCI, serious cardiovascular complications are a major concern, such as unstable blood pressure control related to episodes of orthostatic hypotension and autonomic dysreflexia (AD), characterized by critical blood pressure rises and associated bradycardia [23–25]. Benefits of improved autonomic self-regulation could therefore be substantial for people with SCI, including reduced cardiovascular morbidity associated with autonomic aetiology, orthostatic instability, severity of AD, and improved cognitive functioning, mental health, pain, vitality and sleep [4, 11–13]. Furthermore, this study may lead to improved understanding of the physiological underpinning of brain-autonomic dysfunction and the influence of top down/bottom up controls related to neuroplasticity [22].

Evidence that autonomic activity changes after a SCI and during autonomic challenges suggests autonomic modulation may be a new frontier for improving neuro-cardiac function in individuals with SCI and may mitigate maladaptive plasticity [11]. Underlying hypotheses are the development of intrinsic spinal rhythmicity within the injured cord or recovery of supraspinal pathways [22], demonstrating that autonomic activity measured by HRV in adults with SCI is imbalanced, very different to able-bodied individuals [26]. For example, groups with tetraplegia and paraplegia both have significantly reduced low frequency (LF) HRV activity and impaired sympatho-vagal control [26] and this autonomic imbalance was associated with sleep disorder and fatigue in SCI [4, 26, 27]. It has also been shown that after participating in a 40-min resting/cognitive task, adults with high lesions (at/above T3 level) significantly increased the standard deviation of the average normal-to-normal intervals (SDNN) in the HRV in the LF band (0.04–0.15 Hz) in comparison to an able-bodied cohort [4], suggesting that low frequency autonomic activity in this group is modifiable.

Dysfunctional brain/neural activity is also reported after SCI, in terms of altered brain activity and connectivity [28–32], cerebral blood circulation [33] and cognitive impairment [9, 10]. Research has shown that brain activity is related to HRV activity, clarifying mechanisms of heart-brain neurovisceral relationships [28, 29]. For example, in non-SCI samples, HRV biofeedback improved cognitive and brain activity [29] while increases in the 8–12 Hz electroencephalography (EEG) range (called alpha activity) resulted in changes in HRV [28]. Additionally, adults with SCI have increased risk of thalamocortical dysrhythmia, that is, brain activity characterized by low frequencies (theta/low alpha) similar to aged brain activity in frailty [30, 31]. Furthermore, it is important to improve regulation of cerebral blood flow to compensate for the life-threatening variations in the movement of blood through the circulatory system associated with SCI [33]. It is therefore feasible that HRV self-regulation therapy could result in improved cerebral blood flow, EEG activity, brain metabolism and cognitive capacity in adults with SCI.

The primary objective of this research is to establish the effectiveness of a self-regulation intervention (HRV-F) to improve autonomic/neuro-cardiac function in adults with SCI, that is, improved autonomic regulation and brain/neural activity, such as cerebrovascular circulation and brain connectivity. Secondary objectives involve determining whether HRV-F results in improvements in associated functions such as mental health, cognitive capacity, vitality/fatigue, pain and sleep. While there is limited evidence that HRV-F will achieve these objectives in adults with SCI [12, 21], the scientific basis for our objectives is compelling. This includes: (i) the fact that a healthy autonomic nervous system is of vital importance for daily functioning given it regulates the respiratory, cardiovascular, digestive, endocrine and neural systems [32]; (ii) we have demonstrated functional/adaptive changes in autonomic activity in adults with chronic SCI (i.e., increased HRV) [4]; and (iii) systematic review and meta-analytic evidence concluded HRV-F is an effective self-regulatory intervention for conditions such as depression, heart disease and other clinical conditions in non-SCI populations [15, 18, 34, 35]. This research will address evidence gaps about improving autonomic/neural function in people with SCI, especially in higher and complete lesions. It will also develop a strategic plan for translating findings to those individuals with SCI living in the community with disability and at-risk from secondary conditions like orthostatic instability and AD.

## Methods

### Study design

The design is a two-arm parallel, intention-to-treat, prospective single-blind randomized controlled trial (RCT).

### Objectives

#### Primary objectives

To establish the effectiveness of a self-regulation intervention (HRV-F) to improve autonomic/neuro-cardiac function in adults with SCI. Autonomic/neuro-cardiac function includes autonomic regulation and brain/neural activity, such as cerebral vascular perfusion and brain connectivity.

#### Secondary objectives

1. To determine whether HRV-F results in improvements in associated secondary conditions, such as mental health, cognitive functioning, fatigue, pain, and sleep.
2. To conduct a translation implementation study composed of interviews and a readiness to change survey with a broad range of stakeholders.

### Participants

Participants in the RCT will include 120 adults with a chronic SCI living in the community. Inclusion criteria include: (i) aged 18–70 years with SCI living in New South Wales (NSW), Australia; (ii) English speaking; (iii) traumatic/non-traumatic aetiology with complete/incomplete lesions; (iv) at least 12-months post-injury. Exclusion criteria consist of: (i) evidence of severe cognitive impairment as determined by a neurocognitive screen; (ii) evidence of severe respiratory disorder or impaired respiratory function; (iii) evidence of severe psychiatric disorder, such as bipolar disorder or psychoses, determined by psychiatric assessment, and (iv) taking prescribed ß-blockers. Participants for the implementation study will include up to 100 stakeholders and health professional participants. Stakeholders include individuals from relevant government departments and SCI advocacy groups. Health professionals will include those involved in rehabilitation in the hospital and community settings. Participant recruitment for the implementation study will involve convenience sampling through the NSW Agency for Clinical Innovation State SCI Service (SSCIS) Network and personal contacts in relevant health settings.

### Procedure

The RCT will involve the 120 adults being randomly allocated to either a HRV-F group or a no-treatment control group. Both groups will be similarly assessed over a period of 12-months, that is, at baseline, 10 weeks post-baseline (immediately after the HRV-F treatment for the intervention group), and at 6 and 12-month post-baseline. To test the feasibility of the RCT, the first 10 RCT participants with SCI who meet inclusion and exclusion criteria (5 in the HRV-F and 5 in the control arm) will be monitored for adherence to the HRV-F therapy and assessment, and for any barriers to the intervention and control group membership. The implementation study will involve conducting a mixed methods approach (qualitative and quantitative) to establish how best to translate HRV-F interventions into the community.

### Data management

After recruitment, participants will be de-identified and given a generated code. All data will be stored on a secure online platform called Research Electronic Data Capture (REDCap) [36]. Participants will enter their responses to the online interview/assessment using their personal REDCap link after being given instructions on how to complete. Participants not wanting to enter responses online will complete by face-to-face or telephone assessment with a member of the research team, who will then enter the data onto REDCap. The implementation phase participants will also be de-identified and data from the surveys and interviews will be entered into REDCap for further analysis.

### Randomization and blinding

**Fig. 1:**
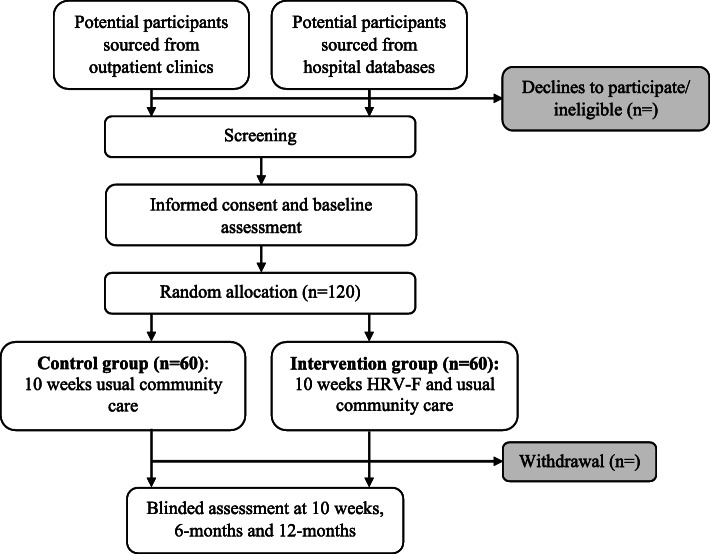
Showing participant allocation to the HRV-F versus Control conditions

RCT participants will be randomized to either the HRV-F group (*N* = 60) or to the no-treatment control group (N = 60) following a stratified randomization procedure. The randomisation allocation ratio is 1:1, while controlling for covariate level of injury (at and above T3 versus below T3). The randomization sequence will be computer generated via RAND function in Microsoft Excel by an independent person and then be uploaded into REDCap. Once the participant is recruited and completed the baseline assessment, then the assessor will inform the team members who are conducting the intervention. The researcher will then randomize a participant via pre-defined randomization sequence in REDCap (see Fig. 1). Team members responsible for recruitment, assessment and analysing the data will be blinded to group allocation. However, the researcher conducting the intervention as well as the participants will be aware of group allocation given it is not possible to blind them to the HRV-F versus control conditions.

### Setting

Participants living in the community will be recruited from outpatient clinics of the two participating sites, that is, Royal North Shore Hospital (RNSH) and Royal Rehab, NSW, or they will approach the research team after viewing study advertisements posted at targeted social and commercial media outlets. Assessment and the HRV-F intervention will only be conducted at the RNSH campus in which the testing laboratory, a dedicated air-conditioned laboratory, is situated. Participants in the HRV-F group will also complete some treatment components in their home setting. The implementation phase participants from the SSCIS Network will be asked to complete an online survey. The SSCIS Network members include clinicians, consumers, government policymakers and representatives from non-government organisations. Ten semi-structured interviews will be conducted via telephone with health professionals who have delivered the HRV-F intervention, or who would deliver the HRV-F if it was introduced into standard practice.

### HRV-F protocol and rationale

#### Rationale

HRV-F is designed to improve autonomic and neurocardiac functioning using computer-based feedback of HRV and controlled diaphragmatic slow breathing around 3–7 breaths per minute. One goal of HRV biofeedback is to stimulate the baroreflex and enhance respiratory sinus arrhythmia (RSA), which involves fluctuations in heart-beat intervals linked with respiration, associated with variations of the parasympathetic cardiac signal [37]. That is, cognition and brain neural activity to adhere to a paced respiration rate and depth, evoking resonant frequency changes to RSA in LF HRV and Total HRV power (in the spectral bands). RSA amplitude (peak-to-trough heart rate difference across the breathing cycle) is contingent with slow breathing [37] and establishing resonant breathing frequency (generally lying between 4 and 7 breaths per minute), identifies the breath rate that is associated with largest gains in RSA and baroreflex activity [37]. Slow breathing rates, and especially breathing at the resonant breath rate, optimally stimulates the baroreflex, resulting in increased RSA and HRV, which is believed to be associated with healthy autonomic and cardiovascular systems [37].

#### Resonant frequency

**Fig. 2:**
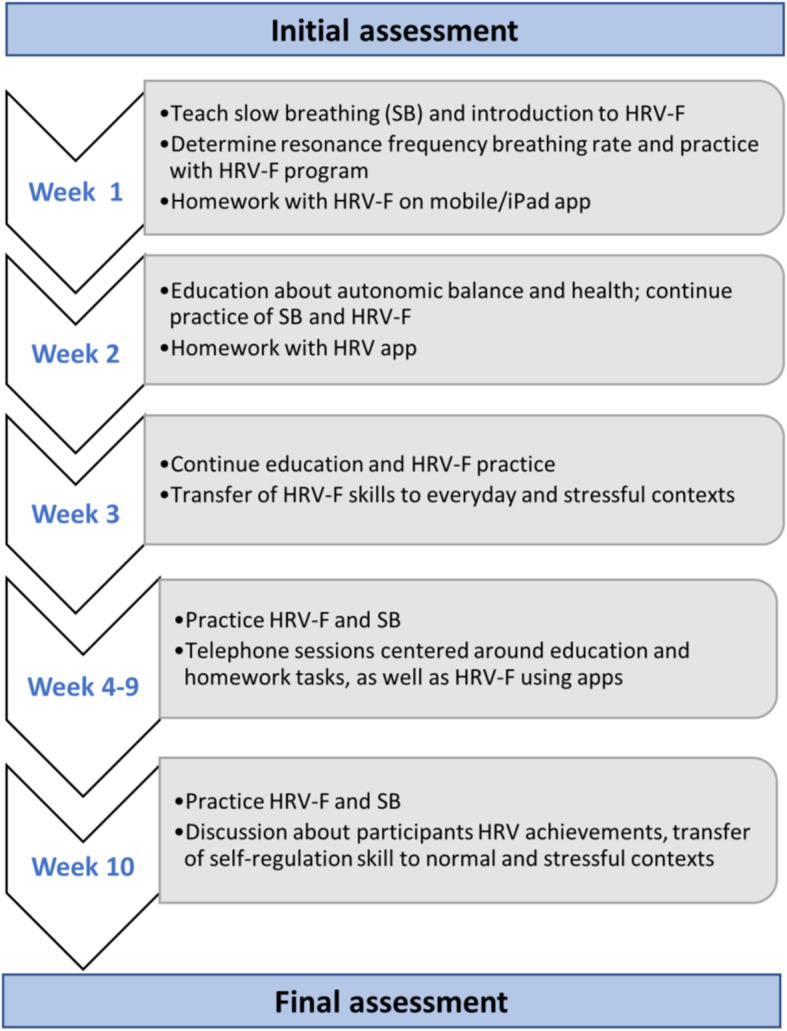
Shows flow of HRV-F intervention sessions over 10 weeks

A central goal of HRV-F, therefore, is to establish a participant’s resonant breath rate [14, 19, 37]. Participants utilize feedback of their HRV in exercises to control their autonomic activity using dedicated biofeedback technology and software (ProComp 2 HRV Systems; Thought Technology, Ltd., Canada, www.thoughttechnology.com). In HRV-F, participants initially use this feedback technology to determine their unique resonant breathing rate, which is established over a period of 15 min while reducing their breath rate by 0.5–1 breaths per minute (BPM), and depending on a participant’s initial resting BPM, gradually decreasing BPM down to 4 or 5 BPM over the 15-min. Once participants have identified their unique resonant breathing frequency, they then proceed to perform varied resonant breathing exercises over the 10-week period. The protocol is shown in Fig. 2.

#### HRV-F training

Ten sessions of formal HRV-F training will be completed over the 10-week intervention. These will involve a mixture of six face-to-face formal sessions (2-h duration each) in the research laboratory and four online/telephone sessions (at-home sessions, up to 1-h duration). Face-to-face HRV-F sessions will be conducted in groups of three participants. However, it is expected that individual sessions will occur given risk of sickness in this population. Self-management strategies will be introduced throughout the 10 weeks of the program, including self-monitoring of mood, anxiety, breath rate, pain/fatigue level and sleep quality, as well as visualisation strategies to be used in conjunction with resonant breathing exercises.

#### Home practice

Homework related to HRV-F will be assigned and a diary used by participants to log homework activities. Homework activities will include (i) resonant breathing exercises as taught in the face-to face sessions; (ii) measuring HRV each day of the 10 weeks using phone-based apps; (iii) self-monitoring of mood, anxiety, breath/heart rate, pain, fatigue and sleep; (iv) maintaining a log of homework activities, and (v) visualisation strategies to enhance their self-regulation of HRV and breathing. Participants will be asked to practise resonant breathing exercises in the home environment, twice a day for around 20 min per session and once a week over the 10 weeks, a team member will provide telephone support. Phone apps will be used to record HRV data and determine adherence, as well as daily/weekly progress. All HRV-F participants will be given a total of $200 Australian in the form of eGift cards throughout the treatment program ($50 at initial and $50 at 10 weeks) and a final $100 after the 12-month assessment.

### Control group protocol

Control participants will receive the same assessment regime as the HRV-F participants. Controls will receive reading material and a brief telephone call each week in the 10-week period to establish their continued participation. Controls will receive similar payment to the intervention group.

### Implementation phase

**Fig. 3:**
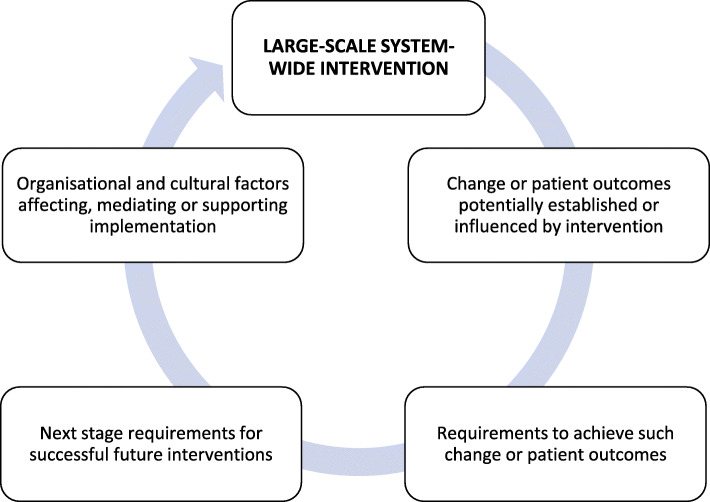
Showing the Implementation at-scale model

Guided by the Theoretical Domains Framework (TDF) [38], the implementation phase participants will be asked to complete an online survey using REDCap to identify facilitators and barriers to the introduction of HRV-F into standard practice. Survey responses will be used to establish how best to translate the HRV-F intervention into healthcare practice for adults with SCI. Survey participation will be voluntary and anonymous. Semi-structured interviews will be conducted via telephone with health professionals who have delivered the HRV-F intervention, or who would deliver the HRV-F, if it was introduced into standard practice. This will include professionals such as psychologists, physiotherapists, nurses, rehabilitation physicians, social workers and occupational therapists. The semi-structured interviews will be used to reflect on the facilitators and barriers identified during the survey and to review the practicality and acceptability of potential solutions to identified barriers. The participants who completed the HRV-F will also be asked two open-ended questions after they complete HRV-F:

“What have been the main challenges for you with learning and doing your therapy?” and “If there was one or two things that could be done differently to improve your therapy, what would they be?” The implementation at scale model to support systems-level diffusion (see Fig. 3), will also inform this work [39]. Articulating a robust implementation strategy addressing challenges faced by health professionals and stakeholders will streamline future translation, preventing costly trial and error attempts in implementing HRV-F into practice.

#### Socio-demographic, injury and medical assessments

Socio-demographic assessment includes sex, age at the time of initial measurement, level of education and employment history. Injury details include age at the time of the SCI, time (years) since the SCI, level and extent (completeness) of the injury using the International Standards for Neurological Classification of SCI (ISNCSCI; https://asia-spinalinjury.org/isncsci-2019-revision). Medical assessment includes medical history (e.g., pre and post-injury health and psychological status), current medications, body mass index assessed from self-reported height and weight, and classified according to World Health Organization guidelines: < 18.5 kg/m^2^ (underweight), 18.5–24.9 kg/m^2^ (normal), 25–29.9 kg/m^2^ (overweight), ≥30 kg/m^2^ (obese).

### Physiological assessment

See Table 1 for all primary and secondary outcome measures and endpoints (baseline versus 10 weeks, 6 and 12 months) for the RCT and implementation phase. The physiological assessment protocol includes:

(i) Activity #1: 5-min resting baseline (spontaneous breathing) after 2-min habituation;
(ii) Activity #2: 5-min Stroop test;
(iii) Activity #3: 5-min recovery (spontaneous breathing);
(iv) Activity #4: 5-min breathing task (paced breathing at around 6 breath/minute);
(v) Activity #5: 5-min recovery (spontaneous breathing).

**Table 1:** Schedule of activities and measures for the RCT and implementation study. All psychometric instruments are scientifically validated and reliable

PROJECT STAGE *(number of participants)*			RCT (***N*** = 120) Pilot and main study phase)						Implementation phase (***N*** = 100)
**TIMELINE/PHASES***(D- day, W: week; M: month; Ph: phase)*			**-7D to 0D**	**0D**	**10 W**	**6 M**	**12 M**	**Fortnightly**	**Ph-1 to Ph-5**
**RECRUITMENT**	**ACTIVITIES**	**Mode of data collection**							
Screening	Eligibility checklist	In-person	**✓**						
	ISCoS cardiovascular function dataset	In-person	**✓**						
	ISCoS pulmonary function basic data set	In-person	**✓**						
Consent procedure	Informed consent procedure	In-person	**✓**						
**PARTICIPANT DESCRIPTORS**	**ASSESSMENTS**	**Mode of data collection**							
Socio-demographics	Socio-demographics details	In-person or online		**✓**					
Pre-injury details	Employment history and psychological status	In-person or online		**✓**					
Medical history	Self-report past/present health history	In-person or online		**✓**					
Injury characteristics	Level of lesion, AIS and FIM	Medical records		**✓**					
Pulmonary function	Pulmonary function test	In-person		**✓**					
**ALLOCATION**	**DESCRIPTION**	**Mode**							
Intervention group	HRV-F + Usual care	In-person and online							
Control group	Usual care	Online							
**PRIMARY OUTCOMES**	**OUTCOME MEASURE**	**Mode of data collection**							
Autonomic function	ECG/ HRV Time domain – Short term	In-person		**✓**	**✓**				
	ECG/ HRV Frequency domain – Short term	In-person		**✓**	**✓**				
Cerebral basal artery blood flow	Doppler assessment – Short term	In-person		**✓**	**✓**				
**SECONDARY OUTCOMES**	**OUTCOME MEASURE**	**Mode of data collection**							
Autonomic function	ECG/ HRV Time domain – Long term	In-person		**✓**		**✓**	**✓**		
	ECG/ HRV Frequency domain – Long term	In-person		**✓**		**✓**	**✓**		
	ADFSCI questionnaire	In-person		**✓**	**✓**	**✓**	**✓**		
Cerebral basal artery blood flow	Doppler assessment – Long term	In-person		**✓**		**✓**	**✓**		
Neurophysiological function	Continuous BP	In-person		**✓**	**✓**	**✓**	**✓**		
	EEG	In-person		**✓**	**✓**	**✓**	**✓**		
	NIRS	In-person		**✓**	**✓**	**✓**	**✓**		
	EOG	In-person		**✓**	**✓**	**✓**	**✓**		
	Skin conductance, BVP, respiration and temp	In-person		**✓**	**✓**	**✓**	**✓**		
Secondary health conditions	SCI-SCS	In-person and online		**✓**	**✓**	**✓**	**✓**		
Cognitive function	NUCOG	In-person and online		**✓**	**✓**				
	Stroop test	In-person and online		**✓**	**✓**				
Psychological/mental health function	GADS	In-person and online		**✓**	**✓**	**✓**	**✓**		
	PHQ-9	In-person and online		**✓**	**✓**	**✓**	**✓**		
Psychosocial function	ISCoS pain questionnaire	In-person and online		**✓**	**✓**	**✓**	**✓**		
	FSS	In-person and online		**✓**	**✓**	**✓**	**✓**		
	PSQA	In-person and online		**✓**	**✓**	**✓**	**✓**		
	PCS	In-person and online		**✓**	**✓**	**✓**	**✓**		
Personal factors	*CD-RISC2 Short Form*	In-person and online		**✓**	**✓**	**✓**	**✓**		
Quality of life	*EQ-5D-5L*	In-person and online		**✓**	**✓**	**✓**	**✓**		
Participation and disability	*WHODAS domain – participation items*	In-person and online		**✓**	**✓**	**✓**	**✓**		
Consumer perception and satisfaction	Self-reported perception of change and satisfaction	In-person and online			**✓**				
Costs for Economic evaluation	Self-reported direct and indirect costs	Diaries (up to 12 m)						**✓**	
Adverse events	*Self-monitoring of Adverse events*	Diaries (up to 12 m)						**✓**	
Self-monitoring *of mood, breath rate, pain, fatigue and sleep quality*	*Numeric rating scale (0 to 10)*	Diaries (up to 12 m)						**✓**	
**EXPLORATORY OUTCOMES**	**OUTCOME MEASURE**	**Mode of data collection**							
Personal factors	*WHO ASSIST*	In-person and online		**✓**	**✓**	**✓**	**✓**		
	*COPE inventory – brief*	In-person and online		**✓**	**✓**	**✓**	**✓**		
	*ADAPSS – Short-form*	In-person and online		**✓**	**✓**	**✓**	**✓**		
	*MSES*	In-person and online		**✓**	**✓**	**✓**	**✓**		
Psychological/mental health function	PTSD Checklist 5	In-person and online		**✓**	**✓**	**✓**	**✓**		
Psychosocial function	SSQ6	In-person and online		**✓**	**✓**	**✓**	**✓**		
	Berlin Questionnaire	In-person and online		**✓**	**✓**	**✓**	**✓**		
Environmental factors	*Care, family and compensation status questionnaire*	In-person and online		**✓**	**✓**	**✓**	**✓**		
Participation and disability	*RTW Questionnaire*	In-person and online					**✓**		
**IMPLEMENTATION OUTCOME**	**MEASURE**	**Mode of data collection**							
Qualitative/ quantitative assessment	*Stakeholder interviews and surveys*	In-person and online							**✓**

*Abbreviations*: *ADAPSS* Appraisal of DisAbility Primary and Secondary Scale, *AIS* American Spinal Injury Association Impairment Scale, *ADFSCI* Autonomic Dysfunction Following Spinal Cord Injury, *BP* blood pressure, *COPE* Coping Orientation to Problems Experienced, *CD-RISC2* Connor-Davidson Resilience Scale 2 item short form, *ECG* electroencephaolography, *EEG* Electroencephaolography, *EOG* Electrooculography, *EQ-5D-5L* Euro Quality of Life 5-Dimensional 5-level, *FIM* Functional Impairment Scale, *FSS* Fatigue Severity Scale, *GAD-7* Generalized Anxiety Disorder 7-Item Scale, *HRV* heart rate variability, *ISCoS* International Spinal Cord Society, MH, *MSES* Moorong Self-efficacy Scale, *NUCOG* Neuropsychiatry Unit Cognitive Assessment Tool, *NIRS* Near infrared spectroscopy, *PCS* Pain Catastrophizing Scale, *PHQ-9* Patient Health Questionnaire-9, *PSQA* Pittsburgh Sleep Quality Index, *PCL-5* Post-traumatic Stress Disorder Checklist for the Diagnostic and Statistical Manual of Mental Disorders Fifth Version, *RTW* return to work, *SCI* spinal cord injury, *SCI SCS* Spinal Cord Injury Secondary Condition Scale, *SSQ6* Social Support Questionnaire, *Temp* temperature, *WHO ASSIST* World Health Organisation Alcohol, Smoking, and Substance Involvement Screening Test, *WHODAS* World Health Organisation Disability Assessment Scale

### Primary physiological outcome measures

- Electrocardiography (ECG) assessment from which HRV will be calculated at 10 weeks (short-term effect), in order to determine whether HRV-F results in improved autonomic nervous system function. HRV via ECG (electrodes on chest, modified lead II configuration) will be measured using the PowerLab 8/35 8-channel, 16-bit resolution recorder data acquisition system with programmable gain and two galvanically-isolated channels FE232 Dual Bio Amps (ADInstruments, www.adinstruments.com). A sampling rate of 512 Hz will be used for time/spectral analyses [4]. HRV signal processing will be performed using Kubios HRV Premium software (Version 3.3.1, University of Kuopio, Finland). HRV indexes neuro-cardiac function/regulation, generated by bidirectional heart-brain interactions (i.e., top-down influences on cardiac and peripheral physiological processes and bottom-up influences on central control mechanisms) and dynamic non-linear autonomic nervous system (ANS) regulation. HRV reflects the innervation of the heart by the sympathetic/parasympathetic divisions of the ANS [4]. In the time domain, average and standard deviation of the normal-to-normal intervals (SDNN; the variability of the heart) and root mean square of the successive differences (RMSSD, vagally mediated HRV) will be calculated. In the frequency domain, low frequency (LF), high frequency (HF, cardiac vagal control) and total power will be assessed. Details of a HRV protocol we have employed are available [40].

(ii) Functional transcranial doppler sonography (fTCD) to assess cerebral artery blood flow at 10 weeks (short-term effect), to determine cerebrovascular benefits associated with the HRV-F, using Multi-Dop T digital with QL routine software (Compumedics Limited, www.compumedics.com.au). fTCD assesses real-time cerebral blood flow velocities/circulation in basal cerebral arteries of the participants in response to the intervention [41]. fTCD is a reliable method for measuring cerebrovascular changes, provides high temporal resolution and is least susceptible to artefact than other imaging techniques [41].

Electrocardiography (ECG) assessment and Functional transcranial doppler sonography will be performed using the same instruments (as per primary outcomes) at 6 and 12 months (long-term effects).

### Secondary physiological outcome measures

#### Electroencephalography (EEG), electrooculography (EOG), respiration/breath rate, skin conductance, blood volume pulse and finger temperature

To measure these secondary outcome measures, two FlexComp electrophysiological monitoring systems (each having 10-channels each; Thought Technology, Ltd., Canada, www.thoughttechnology.com) with BioGraph Infiniti software, will be used for data acquisition of EEG (11 channels using International 10–20 montage system: F3, Fz, F4, C3, Cz, C4, P3, Pz, P4, O1, O2), skin conductance (2 channels, right and left hand), blood volume pulse (2 channels, right and left hand), EOG (1 channel), respiration (1 channel) and peripheral skin temperature (2 channels, right and left hand). The FlexComp has a sampling rate of 2048 samples/sec with 14 bits of resolution (1 part in 16,364). EEG will be used to assess change in brain wave activity in response to the HRV-F and will be spectrally analysed using fast Fourier Transform and a Hanning Window. Sophisticated noise/artefact removal strategies will be used to improve noise:signal ratio [31]. EOG will be used as a physiological measure of fatigue [40]. Skin conductance, blood volume pulse and finger temperature will assess sympathetic arousal and respiration breath rate [42].

#### Near infrared spectroscopy

The Brite MKII 27-channel functional near infrared spectroscopy (fNIRS) system (Artinis Medical Instruments B.V., Amsterdam, Netherlands) will be used to quantify brain oxygenation and neurological activation associated with the HRV-F intervention and complement the EEG measures. NIRS data analysis will be via the OxySoft (Artinis Medical Instruments B.V., Amsterdam, Netherlands) dedicated software used to collect, store, view, and analyse all NIRS signals [43].

#### Continuous blood pressure

Human NIBP Nano Foundation System (ADInstruments, www.adinstruments.com) will be used to record continuous blood pressure (BP) via non-invasive dual finger cuff system (Labchart software). This will ensure measurements of systolic and diastolic BP as well as BP variability (BPV), to quantify cardiovascular benefits associated with the HRV-F. Time series and spectral analysis of BPV and HRV will be used to evaluate baroreflex function/sensitivity and overall (cardiovascular) autonomic balance, with LF HRV indexing sympathetic activity and HF-HRV indexing cardiac vagal control.

#### Assessment protocol

Primary and secondary physiological outcomes will be simultaneously recorded with participants sitting in their wheelchair, according to the following protocol: (i) resting phase of 5 min (after 2 min of habituation); (ii) mental challenge task (e.g. Stroop test) of 5 min; (iii) recovery phase of 5 min (resting and spontaneous breathing); (iv) breathing task of 5 min (paced breathing at 6 breath/min); 5-min recovery of 5 min (resting and spontaneous breathing). To control circadian influences, assessment will occur in the mornings (9 am-1 pm) and will be conducted in a quiet temperature-controlled room in the research laboratory. Participants will be asked to refrain from alcohol, caffeine and smoking at least 12 h before the assessment and to empty their bladder at the arrival to the laboratory.

### Secondary outcome measures- self-report (online) interview/assessment

#### Autonomic dysfunction Follgeowing spinal cord injury (ADFSCI)

This questionnaire is a measure of self-reported frequency and severity of symptoms during hypo- and hypertensive episodes [44]. It has been shown to correlate with blood pressure (BP) instability and to have acceptable test-retest reliability [44].

#### Spinal cord injury secondary conditions scale (SCI-SCS)

SCI-SCS assesses secondary physiological conditions that are associated with SCI using a 4-point ordinal 16-item scale ranging from 0 (not experienced/insignificant problem) to 3 (significant/chronic problem) [45]. Participants rate how these conditions have affected their activities and independence in the last 3-months. Examples of secondary conditions include pressure ulcers and bladder dysfunction. Higher scores indicate more significant impact [45].

#### Cognitive assessment

The Neuropsychiatry Unit Cognitive Assessment Tool (NUCOG) [46, 47], a neurocognitive measure validated for SCI, will be used to assess cognitive functioning using age-adjusted normative scores from a scoring manual. The NUCOG domains include: attention, visuoconstructional ability, memory, executive, and language. Items needing hand function (e.g., object reproduction) will be modified as in prior studies where this has been demonstrated not to alter validity of NUCOG scores [47]. Where it is not possible to administer NUCOG assessments face-to-face (e.g., due to social distancing restrictions due to COVID-19), administration will occur by online methods [10].

#### Generalised anxiety Disorder-7 (GAD-7)

GAD-7 is a seven-item psychometric tool that assesses severity of generalized anxiety and probable generalized anxiety disorder (GAD) [48]. Items request individuals to rate severity of anxiety symptoms experienced over the prior 2 weeks. High scores represent more severe generalized anxiety symptoms. GAD-7 has demonstrated reliability and validity [48].

#### Patient health Questionnaire-9 (PHQ-9)

PHQ-9 assesses nine Diagnostic and Statistical Manual of Mental Disorders (DSM) criteria for depressive disorder over the prior two-week period [49]. Items range between 0 ‘not at all’ to 3 ‘nearly every day’, with higher scores indicating more severe depressive mood. PHQ-9 has demonstrated reliability and validity [49].

#### International spinal cord injury basic pain dataset

This includes four numerical rating items ranging from 0 (no pain) to 10 (extreme or worst pain) for pain intensity and pain interference in day-to-day activities, mood and sleep [50]. Numerical pain rating scales have demonstrated acceptable test-retest reliability and validity [51].

#### Fatigue severity scale (FSS)

The FSS is a 9-item self-report scale that measures fatigue and its influence on lifestyle and function [52]. Items are scored on a 7-point Likert scale (1 = strongly disagree and 7 = strongly agree). Higher scores suggest more severe fatigue. The FSS has demonstrated validity [52].

#### The Pittsburgh sleep quality index (PSQI)

PSQI measures sleep quality across seven domains: subjective sleep quality, sleep latency, sleep duration, habitual sleep efficiency, sleep disturbances, use of sleep medication, and daytime dysfunction [53]. Scoring employs a 0–3 Likert scale, with high scores indicating poor sleep. The PSQI has demonstrated validity [53].

#### Pain catastrophizing scale (PCS)

The PCS is a cognitive bias self-report scale consisting of 13-items that measure degree of catastrophizing type thinking a person is using (e.g., “I can’t stop thinking about how much it hurts”). Items are scored 0–4, with a total score of 54. Higher scores suggest more significant cognitive bias/pain catastrophizing [54]. The PCS has been shown to be reliable and valid [54].

#### Connor-Davidson resilience scale (CD-RISC2)

CD-RISC2 is a 2-item brief version of the 25-item CD-RISC. It measures perceived resilience over the past month with higher scores reflecting greater resilience [55]. The two items are ‘able to adapt to change’ and ‘tend to bounce back after illness or hardship’. The CD-RISC2 has demonstrated internal consistency, test-retest reliability, convergent validity, and divergent validity, and correlates well with the full scale [55].

#### EUROQOL version 5D-5L (EQ-5D-5L)

EQ-5D-5L includes five dimensions about aspects of health, that is, mobility, self-care, usual activities, pain/discomfort, and anxiety/depressive mood [56]. Dimensions have five levels of response, from “no problems” to “extreme problems”. Participants are asked to indicate the most appropriate response-level for each of the five dimensions. The EQ-5D-5L has demonstrated validity [56].

#### World Health Organization disability assessment schedule-participation domain (WHODAS-2)

The WHODAS-2 is disability assessment instrument based on the International Classification of Functioning, Disability, and Health (ICF) [57]. It provides a global measure of disability and 7 domain-specific scores. Only the Participation domain is being assessed. The WHODAS-2 has demonstrated validity [57].

#### Self-reported satisfaction with health services received and change in health

Two in-house Likert scales that assess the participant’s perceived satisfaction with received health services and their change in their health over the past 3 months.

### Health-related cost, adverse events and monitoring of the mood, breath rate, pain, fatigue and sleep quality

These variables will be measured using a fortnightly participant’s diaries.

### Exploratory outcome measures- self-report (online) interview/assessment

#### Post-traumatic stress disorder checklist short-form (PCL-5-SF)

PCL-5-SF is a 4-item version of the 20-item PCL-5. This abbreviated version has demonstrated reliability for detecting symptoms of post-traumatic stress disorder (PTSD) [58]. Participants are asked to rate symptoms experienced over the past 4 weeks including avoidance, negative changes in cognition and mood, and changes in arousal and reactivity. This is done on a 5-point Likert scale from ‘not at all’ to ‘extremely often’. Higher scores suggest more severe traumatic stress [58].

#### Brief coping orientation to problems experienced (brief COPE)

Brief COPE is a 28-item self-report questionnaire assessing ways of coping with stressful life events [59]. Only questions 2, 3, 7, and 8 of the Brief COPE will be included to minimize assessment time. The four items assess ‘Active – Approach’ and ‘Avoidant – Denial’ coping styles [59].

#### Social support questionnaire short form (SSQ-6)

The SSQ-6 is a 6-item questionnaire assessing perceived social support, such as the number of people participants believe are available for social support, and how satisfied they are with the level of support they receive. Items are scored from ‘very dissatisfied’ to ‘very satisfied’ on a 6-point Likert-type scale. The SSQ6 has demonstrated validity [60].

#### Moorong self-efficacy scale (MSES)

MSES measures self-efficacy related to expectations of control about functional activities of daily living in individuals with SCI [61]. Participants are asked to rate perceptions of their belief about their ability to manage 16 tasks on a 7-point Likert scale with 1 being very uncertain and 7 being very certain. Higher scores suggest higher perceived self-efficacy to perform functional activities [61].

#### Appraisals of DisAbility: primary and secondary scale (ADAPSS) short-form

ADAPSS Short-Form measures SCI-specific appraisals using 6 items from the full ADAPSS scale. It has a 2-factor structure of “catastrophic negativity” and “determined resilience”. The ADAPSS Short-form has demonstrated validity [62].

#### Berlin questionnaire (BQ)

The Berlin questionnaire is a questionnaire used to assess obstructive sleep apnea. It involves 11 items about snoring, breathing problems during sleep and fatigue during wake hours [63]. The BQ has demonstrated validity and sensitivity/specificity [63].

#### World Health Organisation alcohol, smoking, and substance involvement screening test (WHO ASSIST)

Question 2 of the WHO ASSIST requests information on the frequency of use of ten substances during the past 3 months, including substances such as tobacco, alcohol, cannabis, cocaine, amphetamine-type stimulants, inhalants, sedatives, hallucinogens, opioids, and ‘other’ drugs [64]. Responses are scored on a 5-point Likert scale, from ‘never’ to ‘daily or almost daily.’ Reliability of the WHO ASSIST has been demonstrated [65].

#### Return to work questionnaire

Return to work will be measured using a return to work questionnaire designed especially for the study with 5-items related to employment nature and status.

#### Environmental factor questionnaire

Care, family and compensation status will be assessed using a questionnaire designed especially for the study with 7-items.

#### Implementation participant survey and semi-structured interviews

Participant surveys will include a mix of positive and negatively phrased closed and open-end questions based on the 14 TDF domains designed to identify facilitators and barriers to the introduction of HRV-F. The semi-structured interviews will ask participants to reflect on the facilitators and barriers identified during the survey and to comment on the practicality and acceptability of potential solutions to identified implementation barriers.

### Economic evaluation

An economic assessment will be conducted that will include costs potentially incurred by participants and healthcare providers. This perspective is required because cost consequences of this study intervention will extend beyond the domain of healthcare. To conduct an economic analysis, the direct and indirect healthcare costs of the intervention and the participants will be acquired from the participant’s cost diaries completed in the study. The economic analyses will include the following:

1. A cost-effectiveness within-study analysis will be conducted. Incremental cost-effectiveness ratios (ICER) will be calculated for the incremental increase in time and frequency domain parameter in the HRV-F group compared with the Control group.
2. A cost-utility analysis to determine incremental cost per quality-adjusted life-year (QALY) gained. The Australian valuation of the EQ-5D will be used to calculate utility weights on a 0–1 scale where 1 represents very good health and 0 represents death. QALYs will be determined by multiplying utility by duration.

Resources will be valued using standard economic evaluation guidelines. Discounting will be set as 5% into the model.

### Excluded medications and treatments

To maintain data validity and ensure participant safety, all participants will remain on their prescribed medications as well as any commonly used assistive technologies that will not interfere with measures such as HRV or EEG. Use of medications will be monitored via a self-report assessment at each time point. Some medications may influence HRV, such as anti-hypertensives and anti-depressants. However, β-blockers have been shown to change HRV substantially [66], participants taking β-blockers will be excluded. In a recent study [26], it was concluded that the majority of medications taken by adults with SCI were not associated with any abnormal influences on HRV data. This included cardiac medications such as ace inhibitors, angiotensin II, anti- platelet, calcium channel blockers, muscle relaxants and anti-convulsants. Even though it is expected that these medications will have some influence on HRV measures, randomization should control any such affects. However, if the data suggests that medication usage is not balanced across the RCT arms, then medication usage will be adjusted for in the statistical analysis to limit potential confounding.

### Control of bias

A number of strategies will be used to manage bias. Intention-to-treat analysis involves all randomized participants being included in the statistical analysis according to the group they were originally assigned, regardless of whether they received all or any of the treatment. Intention-to-treat analysis helps to provide an unbiased estimate of the effectiveness of assignment to the HRV-F intervention. Selection bias will be controlled using concealed randomization and multivariable analysis adjusted for potential confounders such as education and age, as well as stratifying for level of lesion and sex. Furthermore, only adults with a chronic SCI will be recruited into the study and inclusion criteria will be strictly adhered to, so as to reduce confounding of data. Performance bias will be minimized by appropriate blinding to group assignment. Attrition bias addressed by using statistical analyses like linear mixed model analysis with repeated measures, as well as regular follow-up/communication with participants in both groups, the length of the assessments will be kept at a minimum, small financial participation incentives will be used and final results of the study will be made available to all participants.

### Adverse events

HRV-F is a low risk self-regulation intervention. It is unlikely to result in adverse events caused by the intervention or the devices used in the study. To monitor possible adverse events the following will occur: (a) A detailed record of all related and unrelated adverse events will be documented and kept in a locked cabinet or online in a password protected file. (b) The impact of each event on participant safety or on trial conduct will be clarified. (c) Assess and categorise the safety reports received from investigators. (d) Report all suspected unexpected serious adverse reactions. (v) Report to the sponsor (The University of Sydney) within 24-h of becoming aware of any serious adverse events. (vi) Report to the sponsor all safety critical events and any additional requested information relating to reported deaths.

### Power analyses

For the primary outcome measures, assuming an α = 0.05, a small to moderate effect size of 0.3 (based on results from studies in other conditions), with 2 groups (HRV-F and control) of 50 in each group and four assessment periods, estimated statistical power is 0.99 [67]. To allow for reduced participant numbers due to loss to follow-up, sickness, death, or unwillingness to continue, we will recruit up to 120 participants.

### Statistical methods

Descriptive statistics will be generated for all relevant primary and secondary outcomes at all time-points. Linear mixed models for repeated measures analyses will be used to determine the effectiveness of the HRV-F intervention in autonomic function/HRV and cerebral blood circulation in basal cerebral arteries blood flow. Secondary analyses will determine whether improvements occur in factors such as mental health, cognitive function and sleep associated with the HRV-F intervention. All analyses will be adjusted for factors suspected to confound results, such as education and relevant baseline scores. Latent class mixture modelling will be used to determine trajectories for primary and selected secondary measures like anxiety, depressive mood and quality of life. Missing data will be managed using the linear mixed models, for example, through fitting maximum likelihood estimations generating asymptotically unbiased parameter estimates [68]. Data analyses will be performed using packages such as Statistical Package for the Social Sciences (SPSS version 27, SPSS inc., Chicago, Illinois, USA).

For the implementation study, in which qualitative analyses will also be used, the data will be analysed using valid qualitative packages. Grounded theory, an inductive technique of interpreting the recorded data about the perceptions of the HRV-F and its translation, will be the basis for reaching conclusions about how best to implement and translate the treatment.

For cost-effectiveness, descriptive statistics will be calculated to determine costs and QALYs. The robustness of the costing data will be examined through sensitivity analyses. As in standard economic assessments, costs acquired in this study will likely be skewed, so nonparametric bootstrap methods will potentially be used for testing hypotheses tests and interval estimation. A threshold ICER for Australia will be used to assess value for money.

The ICER will be calculated as per equation (eq. 1) below, that is, the incremental cost per increase in time and frequency domain parameters and as incremental cost per QALY gained in the intervention compared to the controls, in the cost-utility analysis.

1 $$ \mathrm{ICER}=\frac{\mathrm{Cost}\ \mathrm{of}\ \mathrm{intervention}\hbox{-} \mathrm{Cost}\ \mathrm{of}\ \mathrm{control}}{\mathrm{Outcome}\ \mathrm{of}\ \mathrm{intervention}\hbox{-} \mathrm{Outcome}\ \mathrm{of}\ \mathrm{control}} $$

Non-parametric bootstrapping will be used to estimate uncertainty around the ICER and results will be presented on an incremental cost-effectiveness plane. We will use 2021 AU$ as the currency and will consequently derive ICERs and cost-effectiveness acceptability curves.

### Data monitoring committee

To guarantee the participants’ safety, an independent Data Monitoring Committee will meet each year of the study to review and evaluate the study data regarding the safety of the HRV-F intervention, the integrity of the data, appropriate study conduct and progress. The committee will consist of three experts who have no conflicts of interests with any of the researchers.

### Ethical approval and registration

The trial received human research ethics approval (2020/ETH02554) on the 2nd December 2020 from the Northern Sydney Local Health District Human Research Ethics Committee. The trial has been registered at Australian and New Zealand Clinical Trial Registry (ACTRN 12621000870853.aspx) on 6 July 2021. SPIRIT guidelines have been followed for this protocol paper.

## Discussion

Dysfunctional autonomic nervous system and brain activity is associated with significant health problems such as cardiovascular disorder, mental health disorder, sleep disorder, fatigue and cognitive impairment [11, 15–17], and is associated with potentially life threatening conditions in SCI [4, 11, 26, 69]. Treatments for improving autonomic function after SCI are limited so it is timely to establish whether a neuro-cardiac self-regulation therapy like HRV-F can result in improved autonomic and brain function post-SCI. Effectiveness will be assessed by primary outcomes like HRV and basal artery blood flow. It will also be important to establish whether HRV-F is associated with improved secondary conditions such as brain activity, fatigue, sleep, pain, cognitive capacity and mental health. If HRV-F is shown to be an effective intervention for those with chronic SCI, it is crucial that this treatment is translated into the community where it can be implemented by health professionals. Consequently, this study will also conduct an implementation mixed methods study to develop guidelines that can be adopted and translated into the community with the ultimate intention of improving the health of individuals with chronic SCI.

## Data Availability

All chief investigators will have access to the final dataset. All datasets used and/or analyzed during the current study are available from the corresponding author on reasonable request.

## Abbreviations

ANS: Autonomic nervous system
SCI: Spinal cord injury
AD: Autonomic dysreflexia
BP: Blood pressure
BPV: Blood pressure variability
fTCD: Functional transcranial doppler sonography
RCT: Randomized controlled trial
BPM: Breaths per minute
SDNN: Standard deviation of the average NN intervals
RSA: Respiratory sinus arrhythmia
EEG: Electroencephalography
EOG: Electrooculography
NIRS: Near infrared spectroscopy
NUCOG: Neuropsychiatry Unit Cognitive Assessment Tool
HRV: Heart rate variability
HRV-F: Neuro-cardiac self-regulation therapy
HF: High frequency
LF: Low frequency
ADFSCI: Autonomic Dysfunction Following Spinal Cord Injury
ISCoS: International Spinal Cord Society
GAD-7: Generalised Anxiety Disorder-7
PHQ-9: Patient Health Questionnaire-9
PCL-5-SF: Post-traumatic Stress Disorder Checklist for the Diagnostic and Statistical Manual of Mental Disorders Version V Short-Form
SSQ-6: Social Support Questionnaire Short Form
PCS: Pain Catastrophizing Scale
FSS: Fatigue Severity Scale
PSQI: The Pittsburgh Sleep Quality Index
BQ: Berlin Questionnaire
SCI-SCS: Spinal Cord Injury Secondary Conditions Scale
WHO ASSIST: World Health Organisation Alcohol, Smoking, and Substance Involvement Screening Test
Brief COPE: Brief Coping Orientation to Problems Experienced
MSES: Moorong Self-Efficacy Scale
CD-RISC2: Connor-Davidson Resilience Scale
ADAPSS: Appraisals of DisAbility: Primary and Secondary Scale Short-Form
EQ-5D-5L: EUROQOL version 5D-5L
WHODAS-2: World Health Organization Disability Assessment Schedule
REDCap: Research Electronic Data Capture
SPSS: Statistical Package for the Social Sciences
TDF: Theoretical Domains Framework
SSCIS: State Spinal Cord Service
